# Review of the psychometric properties and measurement invariance of the Adult Self-Report Scale for ADHD in a sample of employees in Puerto Rico

**DOI:** 10.3389/fpsyt.2025.1702403

**Published:** 2026-02-04

**Authors:** Ernesto Rosario-Hernández, Lillian V. Rovira-Millán, Rafael A. Blanco-Rovira

**Affiliations:** 1 School of Behavioral & Brain Sciences, Clinical Psychology Programs, Ponce, Puerto Rico; 2 Ponce Research Institute, Ponce, Puerto Rico; 3 Social Sciences Department, Universtiy of Puerto Rico, Cayey, Puerto Rico; 4 Industrial/Organizational Psychology Program, Albizu University, San Juan, Puerto Rico

**Keywords:** ADHD, adult ADHD, ASRS-6, functional impairment, measurement invariance, psychometrics, screening, Spanish-speaking populations

## Abstract

This study examined the psychometric properties and measurement invariance of the 6-item Adult ADHD Self-Report Scale Screener (ASRS-6) in a sample of 753 Spanish-speaking employed adults in Puerto Rico. Confirmatory factor analyses supported a bifactor model with a dominant general ADHD factor and two weaker specific dimensions (inattention and hyperactivity). The general factor accounted for substantial shared variance (ECV = .520; ω_H_ = .724), supporting the use of the total score as the primary indicator of ADHD symptom burden. Internal consistency estimates were satisfactory, and scores were significantly higher among participants with a self-reported ADHD diagnosis. The ASRS-6 also demonstrated strong screening accuracy, with an AUC of.91 in ROC analysis. Importantly, ADHD symptoms were associated with functional impairment markers, including lower job satisfaction, greater burnout, reduced work engagement, and higher physical health complaints, underscoring the ecological validity of the screener in real-world settings. Findings support the ASRS-6 as a psychometrically sound and clinically useful tool for adult ADHD screening in Spanish-speaking populations. Its brevity and strong diagnostic performance make it suitable for use in psychiatric and primary care contexts where time-efficient assessments are needed.

## Introduction

Attention-deficit/hyperactivity disorder (ADHD) is a neurodevelopmental condition that frequently persists into adulthood, with global prevalence estimates ranging from 2% to 4% (1). Adult ADHD has been increasingly recognized as a major psychiatric and public health concern due to its strong associations with internalizing and externalizing psychopathology, including depression, anxiety, substance use, and personality disorders (e.g., 2, 3). Beyond comorbidity patterns, ADHD in adults is characterized by pervasive functional impairment, affecting social, academic, and occupational domains (4). Occupational functioning is particularly salient: epidemiological and organizational studies consistently document reduced productivity, increased absenteeism, greater rates of accidents and near-misses, and substantial economic losses attributable to ADHD symptoms in the workforce (5–8). These well-established impairments reinforce the DSM-5 conceptualization of ADHD as requiring not only symptom presence but also significant impairment across major life settings, including work.

Despite its relevance, accurate identification of ADHD in adults remains challenging. Diagnostic evaluation requires integration of retrospective developmental history, collateral reports, and differentiation from symptom presentations of other psychiatric disorders (9). As a result, brief and psychometrically robust screening instruments are essential to efficiently identify individuals who may require a more comprehensive diagnostic assessment. The Adult ADHD Self-Report Scale (ASRS; 8), particularly its 6-item Screener (ASRS-6), is one of the most widely used adult ADHD screening tools. The ASRS-6 was developed using item response theory analyses to retain the most predictive items for DSM-IV ADHD diagnoses and has shown adequate sensitivity, specificity, and cross-cultural applicability (10–12).

However, despite evidence supporting the ASRS in Spanish-speaking populations, important gaps remain. Most Spanish-language validation and adaptation studies have been conducted with outpatient psychiatric or substance use disorder samples, where the ASRS has shown adequate reliability and acceptable sensitivity and specificity (e.g., 10, 13–16). Likewise, other Spanish-language studies have focused on university students or community respondents, emphasizing factor structure, reliability, or prevalence estimates rather than occupational functioning (11, 17, 18). To our knowledge, no published study has specifically examined the internal structure and diagnostic utility of the ASRS-6 in a working adult sample, nor has it systematically linked screener scores to workplace correlates such as burnout, job satisfaction, and engagement, underscoring the need for validation in employed populations.

From a theoretical standpoint, this constitutes a limitation, as multiple occupational health and psychopathology frameworks posit that the cognitive-behavioral features of ADHD—such as deficits in sustained attention, inhibitory control, planning, and time management—are most likely to manifest as measurable impairment in contexts where performance demands are high, structured, and time-sensitive, such as the workplace (4, 19, 20). Job performance models similarly indicate that attentional regulation, error monitoring, and impulse control are central predictors of task performance, safety compliance, and interpersonal effectiveness, domains typically compromised in adults with ADHD (21, 22). A growing body of occupational research shows that adult ADHD symptoms are associated with poorer work outcomes, including higher burnout, reduced task performance, absenteeism, and safety risks (6, 23, 24). Recent findings also indicate that workplace design factors, such as workload, autonomy, and cognitive demands, shape the degree to which ADHD symptoms translate into functional impairment (25, 26). Importantly, research conducted in Puerto Rico has similarly documented that attentional difficulties and work stressors jointly predict occupational strain and diminished well-being among employees (27), underscoring the need to examine ADHD-related functioning in this cultural and organizational context. Together, this evidence highlights the value of validating the ASRS-6 in Puerto Rican workers, as it enables assessment of ADHD symptoms in relation to relevant occupational outcomes within a Spanish-speaking labor force. These theoretical linkages suggest that workplace settings offer an ecologically rich environment in which to evaluate the construct validity and predictive utility of ADHD screening measures.

It is important to note that we do not assume that unemployed adults display different ADHD symptom patterns. Evidence suggests that ADHD symptoms are relatively stable, while functional impairment varies with environmental demands (19, 28). Because workplaces impose high demands on attention, planning, and self-regulation, ADHD-related difficulties tend to be especially evident in this context (4, 7). Thus, our use of organizational variables reflects the relevance of work functioning for adult ADHD, rather than an expectation of different symptom profiles across employment status.

Moreover, ADHD symptom expression does not occur in isolation; it interacts with job design and organizational factors. For instance, high workload, rapid work pace, multitasking, low autonomy, and complex cognitive demands tend to exacerbate ADHD-related functional limitations (25, 28). These interactions imply that the psychometric functioning of ADHD measures may differ across employment contexts, reinforcing the necessity of validation studies that incorporate employed populations rather than general community samples. In the case of Spanish-speaking populations, this need is even more pronounced. Despite high usage of the ASRS in research and clinical practice, empirical data on its structural validity, measurement invariance, and diagnostic accuracy among Hispanic employees remains limited, and no prior study has examined its performance specifically in Puerto Rican working adults.

Given that impairing symptoms often emerge first—or most intensely—in work environments, validating ADHD screening instruments in employed samples enhances ecological validity and strengthens the interpretability of screening outcomes. Importantly, examining associations between ASRS-6 scores and occupational constructs such as task performance, organizational citizenship behavior, counterproductive work behavior, engagement, job demands, and job boredom aligns with contemporary psychiatric and organizational psychology models, which conceptualize impairment as a multidimensional construct observable across psychological, behavioral, and performance indicators (29, 30).

Despite its promise, further validation of the ASRS-6 in Spanish-speaking adult populations is warranted, especially in contexts where psychiatric screening must account for functional impairment in domains such as employment. Importantly, the workplace constitutes a uniquely demanding and structured environment in which ADHD-related impairments become especially visible and clinically meaningful. Unlike other adult settings, work contexts require sustained attention, self-regulation, planning, error monitoring, and adherence to performance and safety standards, capacities that map directly onto the core neurocognitive deficits of ADHD (4, 8). Empirical studies demonstrate that even subclinical ADHD symptoms predict reduced productivity, impaired teamwork, increased safety incidents, and higher rates of absenteeism and job turnover (5–7). As such, the workplace is not only another domain where impairment occurs, but a distinct functional setting in which the consequences of ADHD manifest with organizational, economic, and health implications. This makes work environments particularly relevant, and in many cases essential, for establishing the ecological validity of ADHD screening tools and for identifying adults whose symptoms compromise daily functioning yet remain undiagnosed.

This rationale directly motivates the present study’s focus on employed adults as a crucial yet understudied population for ADHD screening research. Accordingly, the present study evaluates the internal structure, reliability, measurement invariance, and diagnostic utility of the ASRS-6 in a sample of Puerto Rican employed adults. By centering on this population, the study advances a more contextually grounded understanding of ADHD screening in Spanish-speaking workplaces and provides applied evidence supporting the ASRS-6 as a brief, clinically relevant, and occupationally meaningful assessment tool.

## Literature review

Numerous studies have assessed the reliability of the ASRS and its 6-item screener. In general, the full 18-item ASRS shows high internal consistency, Cronbach’s α often around.88-.92 (e.g., 11, 15). For instance, Pedrero Pérez and Puerta García found in a Spanish sample of adults with substance use disorders, the 6-item scale had α = .68 compared to α = .92 for the complete form. Interestingly, Pedrero Pérez and Puerta García removed the last two screener items (which pertain to hyperactivity) raised the alpha to.75, indicating those items contributed least to internal consistency. This finding suggests the inattention items may be more consistently related, whereas the hyperactivity/impulsivity items show more variability, a point to consider in workplace screenings where hyperactive symptoms might manifest differently (e.g. restlessness or impulsivity in meetings). Despite the slightly lower alpha, the ASRS-6 is generally considered reliable for screening purposes. A Peruvian general-population study conducted by Robles et al. (11) with a sample of 4,445 participants reported Cronbach’s α = .81 for the ASRS-6 when treated as a Likert-scale (0-4) summary. Robles et al. also found a test-retest reliability of about *r* = .74 over a 3-week interval for the 6-item screener, demonstrating acceptable stability of scores over time. In practical terms, an employee who screens positive on the ASRS-6 is likely to obtain a similar result if re-screened later, bolstering confidence in the tool’s reliability in organizational contexts.

The ASRS was designed to reflect the two symptom domains of adult ADHD, inattention and hyperactivity/impulsivity, but research has explored whether these scales indeed capture distinct factors or a unidimensional ADHD construct. Evidence to date indicates a somewhat complex structure. Many studies support a two-factor model for the 18-item ASRS corresponding to the DSM symptom groups. For example, using confirmatory factor analysis (CFA) in a Spanish clinical sample, Lozano et al. (14) found the full ASRS fit a DSM-IV two-factor structure (inattention and hyperactivity-impulsivity) well. In the same study, the 6-item ASRS screener was best represented by two correlated factors, essentially separating the four inattention items from the two hyperactivity items. This two-factor screener structure was also observed by Pedrero Pérez and Puerta García (15) in a Spanish addiction treatment sample, where principal component analysis of the 6 items yielded one factor loading the first 4 (inattention) and a second factor for items 5 and 6 (hyperactivity/restlessness), together explaining 62% of variance. Notably, Pedrero Pérez and Puerta García reported that in that analysis the inattention component showed higher reliability (α = .75) than the 2-item hyperactivity component (α = .52), consistent with the lower item-total correlations of the latter items. These findings suggest that while the ASRS-6 does tap a single overarching ADHD construct, it may be worth examining subscale scores (inattentive vs hyperactive symptoms) in research settings or when specific symptom patterns are of interest.

On the other hand, some studies have found a unidimensional or alternative factor structure for the ASRS, especially depending on scoring methods or populations. A Peruvian study (11) found that treating the screener items as a 5-point Likert scale produced a single-factor solution (explaining 53% variance) for the ASRS-6. In contrast, when the same six items were scored in the binary manner (as “positive” or “negative” per each item’s recommended cutoff), an exploratory factor analysis suggested two factors (explaining 52% variance. Robles et al. concluded that the ASRS-6’s apparent factorial structure and the rate of positive screens can vary by scoring method, a reminder that how the instrument is scored (continuous severity vs. dichotomous screening) may influence its psychometric behavior. Meanwhile, a Mexican study conducted by Reyes Zamorano et al. (31) with 540 university students reported a three-factor structure for the full ASRS (18 items) when using exploratory factor analysis. The factors corresponded to inattention (8 items), impulsivity (5 items), and hyperactivity (4 items), together accounting for 49% of variance. This three-factor separation of hyperactivity and impulsivity (rather than combining them) aligns with some models of adult ADHD and is similar to the structure defined in the ICD-10. Indeed, Morin et al. (32) conducted a rigorous factorial study in France applied bifactor modeling to the ASRS and found the best fit was a bifactor model: one general ADHD factor plus three specific factors: inattention, hyperactivity, and impulsivity. In that model, Morin and collaborators concluded that the general factor captured most of the common variance (supporting the use of a total ASRS score), while the specific factors indicated residual groupings of symptoms, with the impulsivity factor being weaker. Overall, these findings across cultures suggest that the ASRS items are multidimensional to some degree. For practical screening in workplaces, the total score or binary classification (positive/negative) is still the primary focus, but understanding the substructure can be useful, for example, an employee might predominantly endorse inattentive symptoms, which could guide tailored interventions (such as organizational strategies for focus and time management).

For a screening tool to be widely applicable, it should function equivalently across different demographic and cultural groups. Reassuringly, research indicates that the ASRS’s structure is largely consistent across sexes, age groups, and languages, with only minor variations. Morin et al. (32) examined a bifactor model with a French sample, the ASRS model showed complete measurement invariance across gender and age groups of adults. Men had higher overall ADHD factor scores than women on average, but interestingly scored slightly lower on the specific hyperactive/impulsive factors than women, a nuance that might reflect reporting differences or true symptom variations. Likewise in Argentina, Scandar et al. (1) found no significant gender differences in ASRS scores and no differences between young and middle-aged adults (18–50 years), supporting the scale’s stability across these groups. Crucially, the ASRS has been tested across many cultures. A recent cross-cultural assessment of the 6-item ASRS v1.1 Screener in 42 countries reported that the instrument has good internal reliability.

(Cronbach’s α =.73, ω = .82 across countries) and partial measurement invariance across different languages, nationalities, and genders (33). In that study, Lewczuk and collaborators found the same two-factor structure of the ASRS-6 in diverse populations, and while some item parameters differed slightly by country/language, the overall construct was comparable. Lewczuk and collaborators indicated that the ASRS-6 achieved invariance up to the level of equal item intercepts (scalar invariance) for most language and country groups, and full metric invariance for gender, with only minor adjustments needed. This means an ASRS-6 score is interpreted similarly whether an employee completes the screener in, say, Spanish, English or Japanese, supporting its use in multinational workplaces or multicultural settings.

There is a growing body of literature specifically validating the ASRS in Latin American samples, which is particularly relevant for Spanish-speaking employee populations. Reyes Zamorano et al. (31) in México was among the first to study the ASRS in Latin American, specifically in México. They validated the Spanish ASRS v1.1 in a sample of Mexican university students and found the Spanish version had high internal consistency (α = .88). Their factor analysis, as noted, yielded three factors (inattention, impulsivity, hyperactivity), and they also reported that women scored higher on the impulsivity factor than men. Supporting the scale’s external validity, the study showed a significant negative correlation between students’ inattention scores and academic performance (semester GPA), indicating that those with more ADHD symptoms tended to have lower grades. The authors concluded that the Spanish ASRS has adequate psychometric properties for use as a screening tool in Mexico. Meanwhile, Peru has contributed epidemiological evidence with the study of Robles et al. (11). Besides the technical findings on factor structure by scoring method, Robles and collaborators demonstrated the feasibility of using the ASRS-6 in a large general population survey and confirmed acceptable reliability and construct validity in a Latin American context. The prevalence of positive ADHD screens in that Peruvian sample varied depending on scoring approach (8.4% with binary scoring vs higher with Likert continuum), but importantly the tool could discern expected associations (e.g. younger adults had slightly higher scores than older, and screening status was related to certain demographic factors). In Argentina, a recent validation study by Scandar (1) with the general population, the ASRS again showed internal consistency and discrimination comparable to the original scale. The study provided local normative data and found that the ASRS could clearly distinguish adults with ADHD from those without (clinical sample vs. volunteers). No significant gender differences emerged, reinforcing that the tool is unbiased for males versus females in this culture. Together, these Latin American studies underscore that the ASRS (both 18-item and 6-item versions) performs well in Spanish-speaking populations, bolstering its use for workplace screening in these regions.

In evaluating a screening tool, it is also important to see how it correlates with other measures and outcomes. The ASRS-6 has shown good convergent validity with clinical diagnoses and other ADHD scales. For example, in Spain, Ramos-Quiroga et al. (10) reported a strong agreement (κ = .88) between a positive ASRS-6 screen and an ADHD diagnosis based on a structured clinical interview (CAADID). In addiction treatment settings, the ASRS-6 has demonstrated convergent validity with longer ADHD questionnaires and even with objective measures; for example, Lozano et al. (14) found that ASRS scores were significantly associated with severity of substance dependence, suggesting a link between uncontrolled ADHD symptoms and worse addiction outcomes. Conversely, discriminant validity has been examined by seeing if ASRS scores remain specific to ADHD symptoms rather than general psychopathology. In a Spanish sample of patients with substance use disorders, Pedrero Pérez and Puerta García (15) noted that while the ASRS-6 effectively identified an ADHD-like impulsivity dimension, it also correlated with measures of personality dysfunction, implying some symptom overlap. They cautioned that in populations with high impulsivity (like addicts), the ASRS might pick up a broader impulsive-compulsive trait not exclusive to ADHD. This highlights the need, especially in occupational health screenings, to follow up positive ASRS results with comprehensive assessments to rule out other causes (e.g. anxiety, substance effects) that can mimic ADHD. Nevertheless, overall evidence for criterion validity is strong, the ASRS-6’s positive screens have been associated with functional impairments (work and academic performance) and respond to known-group differences (e.g. ADHD patients vs controls), as shown in the study of Scandar (1) in Argentina. Such findings support the ASRS-6’s use as a valid indicator of adult ADHD symptomatology in various settings.

The present study aims to examine the psychometric properties of the ASRS-6 in a sample of employees in Puerto Rico. Specifically, we evaluate its internal consistency, factor structure using confirmatory factor analysis, measurement invariance across sociodemographic subgroups (e.g., gender, age, job type), and diagnostic utility through ROC analysis using self-reported ADHD diagnosis. By establishing the psychometric adequacy of the ASRS-6 in this context, our findings will contribute to the cross-cultural validation of the scale and inform its utility for screening adult ADHD in Spanish-speaking occupational settings.

## Method

### Participants

The sample consisted of 753 employed adults who participated in two independent research studies previously conducted by the authors (27, 34). Participants were recruited based on availability in those original studies, which focused on occupational and psychological factors in working populations. Both samples consisted exclusively of employed adults across public and private organizations and included a wide range of occupational roles (e.g., managerial and non-managerial positions), thereby supporting the relevance of focusing on working populations in the present validation. For the purposes of the current study, these data sets were combined and reanalyzed to examine the psychometric properties of the ASRS-6. Therefore, the data analyzed here represent secondary data derived from previously collected samples. Thus, **Table 1** presents the sociodemographic characteristics of the sample. The sample included 34.4% men and 58.0% women. The majority of participants were between the ages of 31 and 50 (45.2%), followed by those aged 21 to 30 (39.2%), and a smaller portion aged 51 years or older (15.3%). Most participants held at least some college education, with 59.5% reporting undergraduate education and 25.9% reporting graduate-level education. In terms of job roles, 80.1% of participants held non-managerial positions, and 69.9% were employed in tenure or permanent positions. Regarding organizational affiliation, 66.8% worked in private sector organizations, while 31.7% were employed in public institutions. The sample was drawn from two distinct studies, with 59.5% from Sample 1 and 40.5% from Sample 2.

**Table 1:** Sociodemographic characteristics of the sample.

Variable	Frequency	Percent
Gender		
Male	259	34.4
Female	437	58.0
Age		
21-30	295	39.2
31-50	340	45.2
≥ 51	115	15.3
Education		
≤ HS	94	12.5
Undergraduate	448	59.5
Graduate	195	25.9
Job position		
Managerial	139	18.5
Non-Managerial	603	80.1
Employment type		
Tenure	526	69.9
Temporary	218	29.0
Organization type		
Public	239	31.7
Private	503	66.8
Study		
Sample 1	448	59.5
Sample 2	305	40.5

n = 753.

### Measures

First, a sociodemographic data sheet was developed and used for the current study. With this, information was collected from the participants related to gender, age, marital status, among other variables, in order to describe the sample of the present study. The additional work-related instruments included in the present study were drawn from the parent research protocols and were selected based on both data availability and theoretical relevance. These measures capture central aspects of occupational functioning and well-being—such as engagement, performance, organizational behaviors, job demands, and boredom—that are closely linked to attentional regulation and self-control, thereby supporting the examination of convergent and criterion-related validity of the ASRS-6.

#### ASRS-6

The Adult ADHD Self-Report Scale-6 item version (ASRS-6) is a brief screening instrument developed by the World Health Organization (WHO) in collaboration with Kessler et al. (8) to detect probable ADHD cases in the adult population. It consists of six items selected from the full 18-item ASRS v1.1 based on their predictive validity. Four of the items assess symptoms of inattention, while the remaining two assess hyperactivity/impulsivity. The instrument has been validated in multiple languages and settings, demonstrating solid internal consistency, construct validity, and diagnostic utility (35, 36). Although the original ASRS-6 response format ranges from 0 = never to 4 = very often, in the present study, items were administered using a Likert scale ranging from 1 (never) to 5 (very often). This decision was based on previous literature supporting the psychometric equivalence and interpretability of the 1–5 format, particularly in Spanish-speaking populations (37, 38). This transformation preserves the ordinal nature of the response options while maintaining the conceptual structure of the original instrument. The Spanish version used in this study corresponds to the validated translation by Ramos-Quiroga et al. (10), which retained the factorial structure and diagnostic sensitivity of the original instrument. The ASRS-6 has been widely used as a valid and efficient screening measure for ADHD in epidemiological, clinical, and occupational contexts, and is recommended when brevity and ease of administration are required (8). A recent study found that reliability fluctuated between.86 and.91 via Cronbach’s alpha (e.g., 39).

#### Work engagement

We used the Utreach Work Engagement (UWES; 40, 41). The UWES is comprised of 17 items measured on a seven-point Likert scale anchored by the response options ‘0’ =never and ‘6’=always. Six items comprised the vigor subscale (e.g., “At my work, I feel busting with energy’). Dedication subscale was measured with five items (e.g., “I find the work that I do full of meaning and purpose”). Finally, the remaining six items comprised the absorption subscale has been reported to fluctuate within.82 to.93 (40). Reliability, using Cronbach’s alpha techniques, of the UWES and its subscales has been reported between.82 to.93 (40). Several studies carried out in Puerto Rico have used it with samples of employed people and its results support the internal structure and its reliability coefficients fluctuated between.81 to.95 using the Cronbach alpha and omega technique (e.g., 42, 43).

#### Task performance

Task performance was assessed using the Task Performance Self-Assessment Scale (TPSAS), this is a five-item instrument developed by Rosario-Hernández et al. (in press) to measure employees’ self-perceived effectiveness in fulfilling core job responsibilities. Items reflect key aspects of task performance, such as adequately completing work duties, complying with formal job requirements, and efficiently performing tasks described in one’s job description. Respondents rated the frequency with which they engage in each behavior using a 5-point Likert-type scale ranging from 1 (*Never*), 2 (*Rarely*), 3 (*Sometimes*), 4 (*Almost Always*), to 5 (*Always*). Higher scores indicate greater perceived task performance. An item example: “I adequately perform the tasks that are required of me as part of my job.” The authors’ scale found support of a unidimensional structure through exploratory and confirmatory factor analyses. In terms of its reliability, the TPSAS obtained a reliability coefficient of.890 using alpha and omega internal consistency techniques.

#### Organizational citizenship behavior

Organizational citizenship behavior (OCB) was assessed using the Organizational Citizenship Behavior Scale, developed and validated by the Rosario-Hernández and Rovira-Millán (2004) (44) following the five-dimensional model proposed by Organ (1988, 1997). The scale consists of 23 items grouped into five theoretically defined dimensions: altruism (e.g., helping colleagues with work-related tasks), courtesy (e.g., preventing problems by keeping others informed), sportsmanship (e.g., tolerating inconveniences without complaining), civic virtue (e.g., participating in organizational affairs), and conscientiousness (e.g., exceeding minimal job requirements). Respondents rated each item on a 6-point Likert-type scale ranging from 1 (*Strongly Disagree*) to 6 (*Strongly Agree*), with higher scores reflecting greater endorsement of organizational citizenship behaviors. In their validation study, authors reported that internal consistency reliability was adequate across all dimensions, with Cronbach’s alpha coefficients of.77 for altruism,.80 for courtesy,.76 for sportsmanship,.82 for civic virtue, and.79 for conscientiousness. Exploratory factor analysis supported the five-factor structure theorized by the authors.

#### Counterproductive work behavior index

Counterproductive work behavior (CWB) was measured using the Counterproductive Work Behavior Index (CWBI), developed and validated by Rosario-Hernández and Rovira-Millán (2008) (45) in a Puerto Rican employee sample. The CWBI assesses the frequency of employee behaviors that intentionally violate significant organizational norms and potentially harm the organization or its members. The final version of the scale consists of 18 items grouped into two subscales: Interpersonal CWB (e.g., spreading rumors or intentionally upsetting coworkers) and Organizational CWB (e.g., stealing company property or sabotaging operations). Participants respond using a 5-point Likert-type scale ranging from 1 (*Never*) to 5 (*Always*), with higher scores indicating greater engagement in counterproductive behaviors. According to its authors, factor analyses supported the two-factor structure of the scale, and internal consistency was high across both subscales and the total scale. Cronbach’s alpha coefficients were.85 for the interpersonal subscale,.87 for the organizational subscale, and.89 for the total score. These findings support the CWBI as a reliable and valid instrument for assessing counterproductive behaviors in workplace settings.

#### Job demands, control and support

We used the Job Demands-Control-Support Model Inventory (JDCMI) developed by Rosario-Hernández and Rovira-Millán (2014). The JDCSMI consists of 29 items rated on a Likert-type scale ranging from 1 (“strongly disagree”) to 6 (“strongly agree”). The instrument includes three major scales: Job Demands, Job Control, and Job Support. The Job Demands scale comprises the subscales of psychological (6-items), emotional (4-items), and physical demands (5-items); the Job Control scale includes the autonomy and skills subscales (3-items each); and the Job Support scale contains the coworker support and supervisory support subscales (4-item each). Both exploratory and confirmatory factor analyses supported its second-order structure, and reliability coefficients ranged from.63 to.95.

#### Job Boredom

To measure underchallenge demands at work, we used the Job Boredom Scale developed by Martínez-Lugo & Rodríguez-Montalbań, (2016) (46). This is an eight-item scale with a Likert seven-point scale ranging from 0 (Totally Disagree) to 6 (Totally Agree). According to authors, confirmatory factor analysis using structural equation modeling support the internal structure of one factor. Also, studies has reported Cronbach’s alpha coefficients fluctuating between.93 to.95 (46), which support the scale reliability.

#### Social desirability

We used the Social Desirability Scale developed by Rosario-Hernández and Rovira-Millán (47). This is a 11-items instrument in a Likert-agreement response format ranging from ‘1’ (Totally Disagree) to ‘6’ (Totally Agree), which pretend to measure a response bias in which people respond to a test thinking what is acceptable socially. Authors report its internal consistency through Cronbach’s alpha to be.86, which is an excellent reliability coefficient. Factor analysis results suggest that the Social Desirability Scale internal structure has only one factor. Moreover, Rosario-Hernández et al. (48) recently reviewed the psychometric properties of the Social Desirability Scale with a sample of 3,855 workers in Puerto Rico after more than 23 years of its development and validation and found the same internal structure and excellent reliability coefficients above.90 using Cronbach’s alpha and McDonald’s omega. Also, they found in their recent study that the scale is invariant among gender, age, job position, type of employment, and organizational type.

### Procedures

The data analyzed in this study were derived from two independent research protocols previously approved by the Institutional Review Board (IRB) at Ponce Health Sciences University. The first dataset was collected under protocol number 130520-ER, and the second under protocol number 1811002688. Both studies were conducted in accordance with the ethical principles outlined in the Declaration of Helsinki and adhered to institutional guidelines for research with human participants. All participants provided informed consent prior to their participation. First, we performed descriptive statistics analyses to obtain sociodemographic characteristics of the sample. Also, we conducted descriptive analyzes of the scale’s items, such as the mean, standard deviation, skewness, and kurtosis. An item analysis was also performed to obtain the discrimination index which is also known as “item-total correlation” or “r_bis_”. Second, ADHD ASRS-6 items were subjected to CFA using the structural equation modeling to examined the internal structure using the weighted least squares-mean and variance adjusted (WLSMV) estimator with the “lavaan” package (49) of the R program version 4.1.2, which robustly deals with potentially non-normal data and items are treated as ordinal (50, 51). To evaluate the fit of our measurement models, we applied a percentile-based approach to interpret the fit indices, as recommended by recent methodological advancements (e.g., 52) given the dichotomous cutoff values are overly simplistic and do not account for nuanced model quality. This approach provides a deeper understanding of model performance by classifying fit indices into percentile-based categories of Very Weak, Weak, Moderate, Strong, and Very Strong fit. Kline (53) recommends the use of at least four fit indices, although more can be reported. One of the indices that is reported is Chi-Square (χ^2^); however, given that the χ^2^ is sensitive to the sample size and therefore the probability of rejecting the hypothesized model increases when the sample size increases, it is recommended to take into account other indices (54) and for this reason it was reported but not taken into consideration as a fit index. Thus we assessed the fit of the models using commonly recommended fit indices: Root Mean Square Error of Approximation (RMSEA), Standardized Root Mean Square Residual (SRMR), Comparative Fit Index (CFI), and Tucker-Lewis Index (TLI). These indices were compared against empirically derived percentile-based ranges to facilitate contextualized interpretation (see **Table 2**):

**Table 2:** Guidelines for model fit interpretation using percentile ranges following Howard et al. (52) recommendations.

Interpretation (Percentile)	Fit Index			
	SRMR	RMSEA	CFI	TLI
Very Weak (<10)	> .100	> .100	< .900	<.900
Weak (10 - 33)	.081 -.100	.081 -.100	.900 -.920	.900 -.920
Moderate (34 - 66)	.061 -.080	.061 -.080	.921 -.950	.921 -.950
Strong (67 - 90)	.030 -.060	.030 -.060	.951 -.980	.951 -.980
Very Strong (>90)	<.030	<.030	>.980	> .980

Third, we assessed measuring invariance across gender, age, education, job position, type of organization, and type of employment. We tested configural invariance, metric invariance, and scalar invariance as suggested by some of the literature (e.g., 55–57). We conducted hierarchical tests for invariance of measurement parameters. First, we examined the configured invariance model or pattern invariance, which imposes no equality restrictions on model parameters. This is a necessary condition for testing invariance by comparing it with other invariance models based on fit indices. Second, we examined the weak invariance model or metric invariance. In this model, the factor loadings are treated as invariant across groups. This ensures that the measures are on the same scale across groups for making valid comparisons. Third, we examined the strong invariance model. This model imposes invariance on both factor loadings and item intercept across groups. This is to ensure the underlying factors can be compared across groups. We capitalized on fit index differences for CFI and TLI, and SRMR (i.e., ΔCFI/ΔTLI ≤−.01, & ΔSRMR ≥.015) reference points as recommended by Cheung and Rensvold (58), who found in a Monte Carlo study that these indices were equally sensitive to all types of invariances. Notably, as the *X^2^* is known to be highly influenced by the sample size (e.g., 59), it was reported but not considered as fit index for the invariance testing. In addition to testing measurement invariance across sociodemographic groups, we examined whether the ASRS-6 functioned equivalently across the two parent samples (Sample 1 vs. Sample 2). A series of multigroup CFA models (configural, metric, and scalar) were estimated using WLSMV and evaluated using recommended ΔSRMR, ΔCFI and ΔTLI criteria.

Fourth, we examined convergent and divergent validity of the ADHD ASRS-6 by correlating it to other supposedly similar and different constructs. Also, we performed descriptive, reliability, and correlation analyses for the ASRS-6 to estimate means, standard deviation, internal consistency via Cronbach’s alpha and McDonald’s omega, standard error of measurement and 95% confidence interval for the scale. Finally, to evaluate the screening accuracy of the ASRS-6 total score, a Receiver Operating Characteristic (ROC) analysis was conducted using SPSS (version 29). As part of the data collection in Study 1 (n = 448), participants were asked a single yes/no question regarding whether they had ever been diagnosed with ADHD by a healthcare professional. This self-reported diagnostic item was used as the criterion variable for the ROC analysis, coded as 0 = no ADHD diagnosis and 1 = ADHD diagnosis. Notably, only 3.3% of participants in this subsample (n = 15) reported having received an ADHD diagnosis. However, based on ASRS-6 scoring, 18.8% of participants (n = 84) were classified as being at high risk for ADHD, 32.1% (n = 144) as being at moderate risk, and 49.1% (n = 220) as being at low risk. The ASRS-6 total score was entered as the test variable. Sensitivity and specificity were calculated across a range of cutoff values, and the area under the curve (AUC) was used as a summary index of discriminative performance. AUC values and their 95% confidence intervals were interpreted following established guidelines, with higher values reflecting stronger screening accuracy.

## Results

### Item-level descriptive statistics and discrimination index

**Table 3** presents the item-level descriptive statistics and discrimination indices (r_bis_) for the six items comprising the ADHD Adult Self-Report Scale (ASRS-6) based on a sample of 753 employed adults. The table includes the frequency and percentage of responses across the five-point Likert scale, as well as the mean, standard deviation (SD), skewness, kurtosis, and item-total correlation (r_bis_) for each item. Items 1 through 4, which assess inattention, and Items 5 and 6, which assess hyperactivity/impulsivity, demonstrated adequate variability in response distributions. The response option “Rarely” was most frequently endorsed across all items, but Items 5 and 6 also showed more spread toward the higher frequency categories (“Often” and “Very Often”), indicating higher average scores on the hyperactivity dimension. All items exhibited acceptable levels of skewness (≤ 1) and kurtosis (within ±1.5), suggesting that the data are reasonably normally distributed for Likert-type responses. Mean item scores ranged from 2.01 to 2.88, with the highest means observed for items measuring hyperactivity (Items 5 and 6). The rbis ranged from.722 to.774, indicating strong internal consistency and good item discrimination. According to guidelines proposed by Nunnally and Bernstein (60), rbis values above.30 reflect acceptable item discrimination, and values above.70, as observed here, indicate excellent item performance. These findings provide preliminary evidence for the measurement quality of the ASRS-6 in this occupational sample.

**Table 3:** Item Univariate analysis and discrimination index (r_bis_) of the ADHD Adult Self-Report Scale (ASRS-6).

Item	Frequencies of response options of ASRS-6					Descriptive statistics				
	Never	Rarely	Sometimes	Often	Very often	M	SD	Skew	Kur	r_bis_
ASRS-1	212	326	145	46	212	2.13	0.995	0.893	0.575	.734
	28.2%	43.3%	19.3%	6.1%	28.2%					
ASRS-2	257	315	115	48	257	2.01	0.983	0.991	0.681	.740
	34.1%	41.8%	15.3%	6.4%	34.1%					
ASRS-3	178	301	187	60	178	2.28	1.025	0.671	0.072	.722
	23.6%	40.0%	24.8%	8.0%	23.6%					
ASRS-4	200	292	176	62	200	2.22	1.027	0.678	-0.005	.726
	26.6%	38.8%	23.4%	8.2%	26.6%					
ASRS-5	125	200	179	140	125	2.88	1.297	0.169	-1.066	.748
	16.6%	26.6%	23.8%	18.6%	16.6%					
ASRS-6	127	200	201	147	127	2.80	1.229	0.176	-0.931	.774
	16.9%	26.6%	26.7%	19.5%	16.9%					

n = 753.

### Confirmatory factor analyses

**Table 4** presents the model fit indices, standardized factor loadings, and ancillary bifactor statistics for the ASRS-6 based on unidimensional, two-factor, and bifactor confirmatory factor analysis models. According to the percentile-based evaluation guidelines suggested by Howard et al. (52), the unidimensional model demonstrated inadequate fit, with a CFI of.955 and TLI of.926 falling below the 50th percentile and an RMSEA of.170 exceeding the 95th percentile, suggesting poor approximation of model parameters to the data. In contrast, the two-factor model showed excellent fit, with CFI = .988 and TLI = .978 both falling within the 90th-95th percentile range and RMSEA = .091 within the 75th-90th percentile range, reflecting acceptable but not optimal error approximation. The bifactor model yielded superior fit, with CFI and TLI both equaling 1.000, exceeding the 95th percentile, and RMSEA = .010 falling below the 25th percentile, indicating an exceptionally close fit to the data. Ancillary bifactor statistics further suggested that although a general factor explained a substantial portion of the common variance (ECV = .520; ω_H_ = .724), specific factors, particularly hyperactivity (ω_HS_ = .561), also contributed meaningful unique variance. These results support a bifactor representation of the ASRS-6 as the best fitting model while acknowledging the strength of the two-factor structure in reflecting specific symptom domains.

**Table 4:** Fit indices by model, factor loadings, and bifactor ancillary statistics of the ADHD ASRS-6.

Item	Model						
	Unidimensional	Two-Factor		Bifactor			
		F_1_	F_2_	GF	F_1_	F_2_	I-ECV
ASRS-1	.763	.780		.589	1.000		.258
ASRS-2	.739	.758		.622	.309		.802
ASRS-3	.727	.740		.814	.026		.999
ASRS-4	.725	.739		.734	.125		.972
ASRS-5	.602		.842	.539		1.000	.225
ASRS-6	.507		.634	.411		.312	.634
Interfactor Correlation		.590					
				
Fit Index							
χ^2^ (*df*)	203.730* (9)	58.217* (8)		5.364^NS^ (5)			
SRMR	.087	.048		.016			
RMSEA (90% CI)	.170 (.150 -.190)	.091 (.070 -.114)		.010 (.000 -.052)			
CFI	.955	.988		1.000			
TLI	.926	.978		1.000			
Ancillary Statistic							
PCU				.530			
ECV				.520			
ARPB				.159			
ω_H_				.724			
ω_HS_					.199	.561	

n = 753, *p<.05, NS = Not Significant; F_1_ = Inattention, F_2_ = Hyperactivity. Loadings fixed to 1.0 correspond to specific factors defined by a single indicator and were constrained for model identification. These parameters do not reflect freely estimated loadings and should not be interpreted substantively.

It should be noted that, in the bifactor model, some specific factors were defined by a single indicator. To achieve model identification, the loadings of these indicators on their corresponding specific factors were fixed to 1.0. As a consequence, the residual variance of these items could not be independently estimated, rendering the specific factor statistically indistinguishable from its observed indicator. This constraint reflects a well-known limitation of bifactor models with single-item specific factors and does not represent an empirically plausible loading (i.e., zero measurement error). Accordingly, these specific factors should not be interpreted as substantively meaningful constructs. Interpretation of the bifactor solution therefore centers on the general ADHD factor and associated bifactor indices, which provide more reliable information regarding the scale’s dimensionality and construct validity.

### Convergent and divergent validity

The average variance extracted (AVE) values for both the inattention (.568) and hyperactivity (.564) subscales exceed the.50 threshold, supporting convergent validity (61). Furthermore, the maximum share variance (MSV;.348) is lower than both AVEs, satisfying Fornell and Larcker’s (62) criterion for discriminant validity, indicating that the subscales are empirically distinct despite being strongly correlated. The average share variance (ASV) value of.174 also being lower than AVE supports additional evidence for discriminant validity. Meanwhile, **Table 5** presents the correlation matrix between the ADHD ASRS-6 and various work-related variables, including work engagement, task performance, organizational citizenship behaviors (OCB), counterproductive work behaviors (CWB), job demands, job control, job support, and boredom at work. The total ADHD score, as well as its two subscales, Inattention and Hyperactivity, were examined. As expected, higher ADHD scores were negatively associated with adaptive workplace outcomes such as work engagement (r = -.088 to -.146), task performance (r = -.130), and OCB dimensions (r = -.122 to −.105). Conversely, ADHD was positively associated with counterproductive work behaviors (r = .189), job demands (r = .116), and boredom at work (r = .340), which supports the theoretical link between attentional difficulties and maladaptive occupational functioning. Notably, inattention consistently exhibited stronger associations than hyperactivity with these organizational constructs, suggesting it may be the more impactful dimension in workplace contexts. Finally, the correlation between the ASRS-6 and social desirability was low and not statistically significant (r = .032, p >.05), indicating that participants’ responses to ADHD symptoms were not meaningfully influenced by a tendency to present themselves in a socially favorable light. Similarly, the subscales of inattention (r = .040) and hyperactivity (r = .009) also showed negligible and non-significant associations with social desirability. These findings provide evidence of discriminant validity for the ASRS-6, suggesting that the instrument captures self-reported ADHD symptoms with minimal contamination from socially desirable responding. This is particularly relevant in workplace settings, where employees may otherwise feel pressure to underreport symptoms to maintain a positive image.

**Table 5:** Matrix correlation of the ADHD Adult Self-Assessment Scale (ASRS) with other variables.

Scale/Subscale	Mean	SD	n	ADHD	Inattention	Hyperactivity
ADHD	10.87	5.16	753	1	.918^**^	.815^**^
Inattention	6.36	3.49	753		1	.521^**^
Hyperactivity	4.51	2.40	753			1
Work Engagement	76.40	19.65	753	-.088^*^	-.146^**^	.019
Vigor	27.91	6.76	753	-.137^**^	-.197^**^	-.010
Dedication	23.37	6.62	753	-.067	-.117^**^	.024
Absorption	25.12	7.62	753	-.048	-.099^**^	.038
Task Performance	22.91	3.18	753	-.130^**^	-.198^**^	.005
OCB	109.22	15.20	753	-.122^**^	-.149^**^	-.049
Altruism	36.80	5.56	753	-.069	-.078^*^	-.038
Conscientiousness	13.87	4.19	753	-.101^**^	-.153^**^	.007
Courtesy	14.92	3.08	753	-.085^*^	-.087^*^	-.058
Sportsmanship	13.62	3.89	753	-.003	-.020	.022
Civil Virtue	30.02	7.40	753	-.105^**^	-.113^**^	-.064
CWB	24.28	8.85	753	.189^**^	.220^**^	.089^*^
Interpersonal CWB	10.37	4.38	753	.169^**^	.183^**^	.099^**^
Organizational CWB	13.91	5.11	753	.183^**^	.223^**^	.069
Job Demands	60.38	13.85	305	.116^*^	.104	.091
Psychological Demands	27.77	5.06	305	.059	-.031	.163^**^
Emotional Demands	13.51	5.27	305	.090	.142^*^	-.017
Physical Demands	19.10	6.75	305	.124^*^	.124^*^	.078
Job Control	28.08	4.97	305	-.191^**^	-.258^**^	-.027
Autonomy	12.48	3.76	305	-.152^**^	-.175^**^	-.064
Skills	15.60	2.50	305	-.151^**^	-.250^**^	.042
Job Support	35.64	8.48	305	-.253^**^	-.277^**^	-.126^*^
Coworkers Support	17.94	4.25	305	-.226^**^	-.277^**^	-.073
Supervision Support	17.70	5.39	305	-.219^**^	-.217^**^	-.141^*^
Boredom At Work	6.99	9.00	305	.340^**^	.418^**^	.105
Social Desirability	25.83	13.38	753	.032	.040	.009

*p<.05, **p<.01.

### Measurement invariance

Measurement invariance across the two parent samples was evaluated using multigroup CFA. The configural model demonstrated excellent fit (SRMR = .054, CFI = .985, TLI = .972), indicating consistent factor structure across samples. Constraining factor loadings (metric invariance) produced negligible changes in fit (SRMR = .057, CFI = .985, TLI = .978). Further constraining thresholds (scalar invariance) also did not meaningfully alter model fit (SRMR = .057, CFI = .985, TLI = .988). All changes in SRMR, CFI and TLI were well within recommended thresholds (ΔSRMR<.015, ΔCFI<.010; ΔTLI<.010), supporting scalar invariance across samples. These results justify combining the datasets for subsequent analyses. **Table 6** presents the results of the multigroup confirmatory factor analysis (MG-CFA) conducted to examine the measurement invariance of the ADHD ASRS-6 across key sociodemographic groups, including gender, age, educational level, job position, type of employment, and organizational sector. For each group comparison, configural, metric, and scalar models were tested using WLSMV estimation. Across all comparisons, configural models demonstrated good baseline fit (e.g., CFI ≥.985; TLI ≥.972; SRMR ≤.054), indicating that the two-factor model of the ASRS-6 was structurally adequate in all subgroups. Changes in fit indices between configural and metric models (ΔCFI ≤ |.003|; ΔTLI ≤ |.006|; ΔSRMR ≤.007) and between metric and scalar models (ΔCFI ≤ |.007|; ΔTLI ≤ |.014|; ΔSRMR ≤.005) were within the recommended thresholds for establishing measurement invariance (58, 63). Furthermore, although some chi-square differences (Δχ²) were statistically significant due to the large sample size, the small deltas in approximate fit indices supported full scalar invariance across all tested groups. These findings suggest that the ASRS-6 provides psychometrically equivalent assessments of ADHD symptoms across diverse employee subgroups, allowing for meaningful comparisons of latent means and relationships in future research and practice.

**Table 6:** Multigroup analysis of the ADHD ASRS-6 by gender, age, education, job position, employment type and organization type to examine measurement invariance.

Model		χ^2^ (*df*)	SRMR	CFI	TLI	Reference Model	Δχ ^2^	ΔSRMR	ΔCFI	Δ TLI	Interpretation
Gender (Male/Female)											
1.	Configural	77.900 (16)	.054	.985	.972	—–					Good baseline fit
2.	Metric	82.332 (20)	.057	.985	.978	1	4.432	+.003	.000	+.006	Metric invariance supported
3.	Scalar	97.170 (36)	.057	.985	.988	2	14.838	.000	.000	+.010	Scalar invariance supported
Age (21-30/31-50/ ≥51)											
1.	Configural	82.303 (24)	.054	.987	.976	—–					Good baseline fit
2.	Metric	97.537 (32)	.059	.986	.980	1	15.234	+.005	-0.001	+.004	Metric invariance supported
3.	Scalar	110.974 (62)	.055	.989	.992	2	13.437	-0.004	+.003	+.012	Scalar invariance supported
Education ( ≤HS/UGS/GS)											
1.	Configural	68.960 (24)	.053	.990	.982	—–					Good baseline fit
2.	Metric	92.590 (32)	.060	.987	.982	1	23.63	+.007	-.003	.000	Metric invariance supported
3.	Scalar	93.434 (62)	.055	.994	.996	2	0.844	-.005	+.007	.014	Scalar invariance supported
Job position (Managerial/Non-Managerial)											
1.	Configural	61.289 (16)	.050	.990	.981	—–					Good baseline fit
2.	Metric	61.331 (20)	.050	.991	.986	1	0.042	.000	+.001	+.005	Metric invariance supported
3.	Scalar	68.610 (36)	.050	.993	.994	2	7.279	.000	+.002	+.008	Scalar invariance supported
Employment type (Tenure/Temporary)											
1.	Configural	73.036 (16)	.052	.988	.977	—–					Good baseline fit
2.	Metric	80.838 (20)	.056	.987	.980	1	7.802	+.004	-.001	+.003	Metric invariance supported
3.	Scalar	90.030 (36)	.054	.988	.990	2	9.192	-.002	+.001	+.010	Scalar invariance supported
Organization type (Public/Private)											
1.	Configural	67.992 (16)	.051	.988	.978	—–					Good baseline fit
2.	Metric	72.180 (20)	.053	.988	.982	1	4.188	+.002	.000	+.004	Metric invariance supported
3.	Scalar	85.664 (36)	.052	.989	.990	2	13.484	-.001	+.001	+.008	Scalar invariance supported

n = 753.

### Reliability and descriptive statistics

**Table 7** summarizes the descriptive statistics, reliability estimates, and confidence intervals for the ASRS-6 and its subscales. The total ASRS-6 score had a mean of 14.32 (SD = 4.52), ranging from 6 to 29 on a possible scale of 6 to 30. The Inattention subscale (4 items) yielded a mean of 8.64 (SD = 3.17), while the Hyperactivity subscale (2 items) had a mean of 5.68 (SD = 2.17). Internal consistency reliability, as estimated by Cronbach’s alpha (α) and McDonald’s omega (ω), was adequate for the total scale (α = .774; ω = .759) and the Inattention subscale (α = .795; ω = .791). The Hyperactivity subscale, consisting of only two items, showed lower reliability (α = .649), and omega could not be computed due to model identification limitations. Standard error of measurement (SEM) values was 2.22 for the total scale, 1.45 for Inattention, and 1.29 for Hyperactivity, yielding confidence intervals of ±4, ± 3, and ±3 points, respectively. These results suggest that the ASRS-6 demonstrates acceptable levels of internal consistency and measurement precision, particularly for the total scale and the Inattention subscale, consistent with prior validation studies (8, 12).

**Table 7:** Descriptive statistic, reliability and 95% confidence interval (CI) of the scores of the ASRS-6.

Scale/Subscale	#Items	Mean	SD	Min	Max	Possible range	Reliability		SEM	95% CI
							∝ (CI)	ω (CI)		
ASRS	6	14.32	4.52	6	29	6 - 30	.774 (.742 -.800)	.759 (.724 -.792)	2.22	±4
Inattention	4	8.64	3.17	4	20	4 - 20	.795 (.764 -.821)	.791 (.756 -.820)	1.45	±3
Hyperactivity	2	5.68	2.17	2	10	2 - 10	.649 (.584 -.703)	###	1.29	±3

n = 753; SD = Standard Deviation, SEM = Standard Error of Measurement, ### = Could not be computed.

### Diagnostic accuracy of the ASRS-6: ROC curve analysis

**Figure 1:**
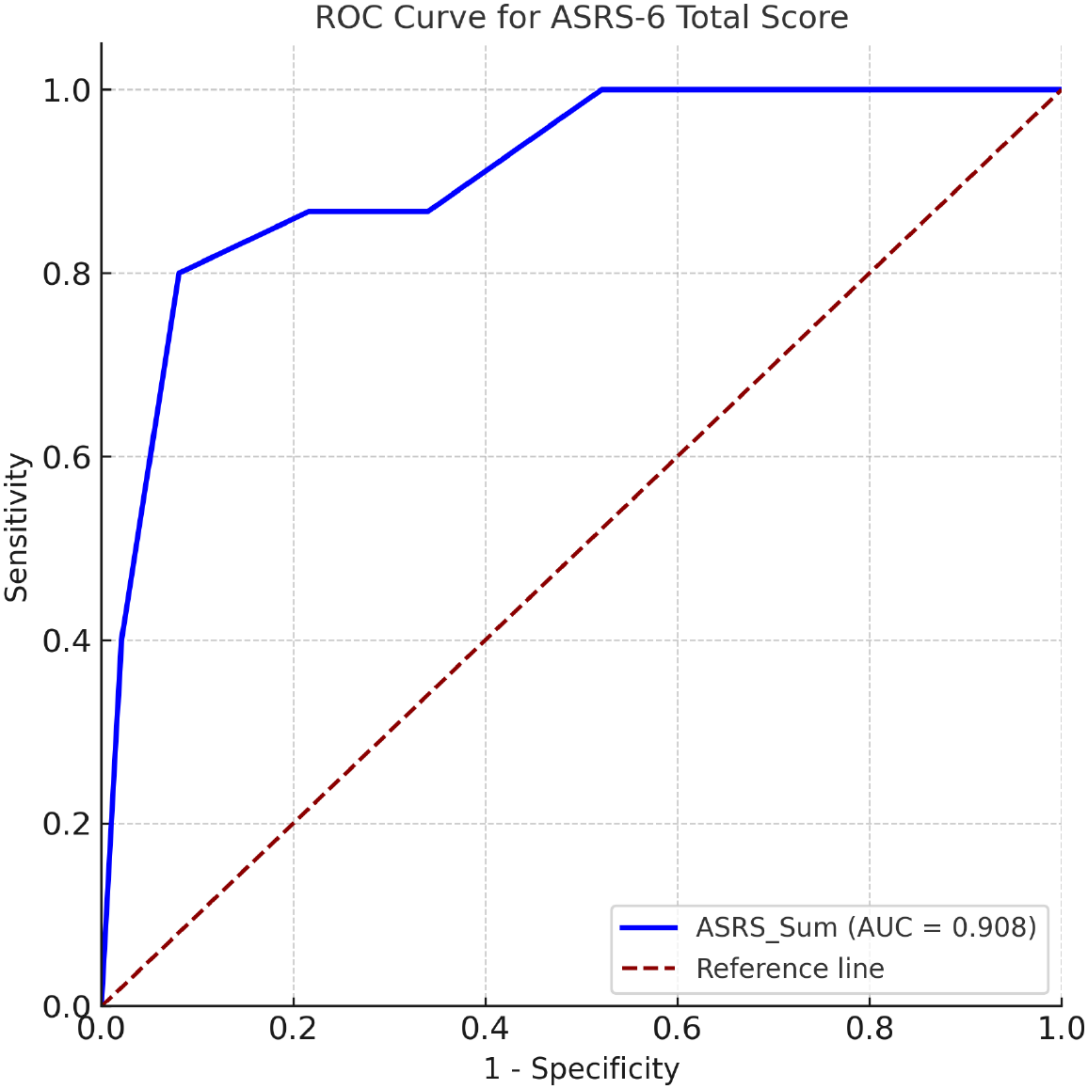
Receiver Operating Characteristic (ROC) curve for the ASRS-6 total score. The ROC curve displays the sensitivity and 1 – specificity across cutoff points for the ASRS-6 total score in predicting clinician-reported ADHD diagnosis. The area under the curve (AUC) was.908 (95% CI [.836,.980]), indicating excellent discriminative performance. AUC = area under the curve.

To evaluate the diagnostic utility of the ASRS-6, a Receiver Operating Characteristic (ROC) analysis was conducted using self-reported ADHD diagnosis as the criterion variable (n = 448 from Study 1). The analysis revealed that the ASRS-6 total score demonstrated excellent discriminative performance, with an area under the curve (AUC) of.908 (95% CI [.836,.980], p<.001; see **Figure 1**). According to established guidelines (64), this AUC value is considered outstanding. Among the tested cutoff values, a score of ≥ 4.5 yielded the optimal balance between sensitivity and specificity. Specifically, this threshold provided a sensitivity of.800 and a specificity of.919, indicating that the ASRS-6 correctly identified 80% of participants who self-reported an ADHD diagnosis, while also correctly classifying nearly 92% of those who did not. These results are consistent with previous validation studies of the ASRS-6 (12, 65), and they support the utility of the instrument as an effective brief screening tool for ADHD symptoms in adult workplace populations.

## Discussion

The present study sought to examine the psychometric properties of the 6-item version of the Adult ADHD Self-Report Scale (ASRS-6) in a sample of employed adults in Puerto Rico. Overall, findings support the ASRS-6 as a valid, reliable, and diagnostically useful instrument for identifying probable ADHD symptomatology in psychiatry and workplace contexts. Our results contribute to the growing cross-cultural literature on ADHD screening tools and confirm the ASRS-6’s suitability for occupational health assessments in Spanish-speaking populations.

Item-level analyses indicated excellent discrimination indices (rbis ≥.72), with item means and response distributions supporting adequate variability. These findings are consistent with prior studies (e.g., 11, 15) and suggest that each item contributes meaningfully to the total score. Internal consistency estimates were adequate for the total score (α = .77; ω = .76) and strong for the Inattention subscale (α = .80). As in past research (e.g., 14), the two-item Hyperactivity subscale showed lower reliability (α = .65), which is expected given its brevity and potential multidimensionality.

Confirmatory factor analysis supported a bifactor structure as the best fitting model, with strong fit indices and ancillary bifactor statistics indicating that both a general ADHD factor and domain-specific dimensions (especially hyperactivity) contribute to the scale’s variance. These findings align with prior work by Morin et al. (32), who emphasized the utility of bifactor models in capturing the hierarchical structure of ADHD symptoms. Nevertheless, the two-factor model also demonstrated acceptable fit and should remain a viable alternative when bifactor modeling is not feasible, particularly in applied settings.

Confirmatory factor analysis indicated that a bifactor structure provided the best overall fit to the ASRS-6 data, supporting the presence of a general ADHD factor alongside more specific symptom dimensions. This finding is consistent with prior methodological work highlighting the usefulness of bifactor models for capturing hierarchical structures in psychological constructs (32). At the same time, the two-factor model demonstrated acceptable fit and may remain a viable alternative in applied settings where bifactor modeling is not feasible. Examination of the bifactor solution revealed a dominant general ADHD factor, with consistently strong loadings across items, indicating that most of the variance is attributable to a common underlying construct. This pattern closely mirrors previous bifactor studies of ADHD symptom measures, including the ASRS and related instruments, which have consistently reported a strong general factor alongside comparatively weaker or less interpretable specific factors (66–71). It is important to note that some specific factors in the present bifactor model were defined by single indicators. To achieve model identification, the loadings of these indicators were fixed to 1.0, rendering the specific factor statistically indistinguishable from its observed item. Such constraints reflect a well-documented limitation of bifactor models with single-item specific factors and do not represent empirically plausible parameter estimates (72, 73). Accordingly, these specific factors should not be interpreted as substantively meaningful constructs. Consistent with current methodological recommendations, interpretation of the bifactor solution therefore centers on the general ADHD factor rather than on the specific factors (74, 75). Taken together, these findings support the conceptualization of the ASRS-6 as a primarily unidimensional screener and reinforce the use of its total score as the most reliable indicator of ADHD symptom burden in adult populations, particularly in applied and occupational research contexts.

In terms of convergent and divergent validity, the ASRS-6 showed a coherent pattern of associations with workplace outcomes, consistent with prior research on adult ADHD. Higher ASRS-6 scores were associated with lower work engagement, poorer task performance, reduced organizational citizenship behaviors, and increased counterproductive work behaviors, with these relationships being more pronounced for inattention (4, 8, 76). This pattern aligns with evidence indicating that attentional difficulties are a primary driver of functional impairment in employed adults with ADHD (8, 77) and mirrors findings from a previous study conducted in a Puerto Rican employee sample, which demonstrated that ADHD symptoms negatively affect task performance and increase counterproductive behaviors both directly and indirectly through reduced work engagement (27).

In addition, higher ASRS-6 scores were related to greater job demands, lower job control and support, and higher levels of boredom at work, further supporting the scale’s convergent validity (28, 30, 78, 79). Divergent validity was evidenced by the negligible association between ASRS-6 scores and social desirability, suggesting that responses were not meaningfully influenced by impression management tendencies (47, 48). Collectively, these findings reinforce the ecological validity of the ASRS-6 as a screening instrument for capturing ADHD-related functional impairment in occupational settings, with inattention emerging as the most influential symptom dimension, consistent with prior adult ADHD research (8, 77).

Crucially, the ASRS-6 demonstrated full scalar measurement invariance across key sociodemographic subgroups, including gender, age, education, job position, employment type, and organizational sector. This finding allows for meaningful comparisons of latent scores across groups and supports the fairness of the ASRS-6 for use in diverse workplace populations. The invariance results are consistent with those reported by Lewczuk et al. (33) and Scandar et al. (1), reinforcing the cross-cultural generalizability of the scale.

The ASRS-6 also showed excellent diagnostic utility. ROC analysis yielded an AUC of.91, indicating outstanding discriminative accuracy (64). A cutoff score of ≥ 4.5 balanced sensitivity (.80) and specificity (.92), closely mirroring results from previous validation studies (e.g., 10, 12). This suggests that the ASRS-6, when administered using a 5-point Likert scale, retains strong diagnostic performance and can be confidently used in brief workplace screenings to identify individuals who may benefit from further ADHD evaluation.

### Theoretical and practical implications

Recent research supports modeling adult ADHD symptoms with a bifactor structure consisting of a strong general factor and weaker specific factors (67, 80). Our results align with this view, showing that the general factor in the ASRS-6 accounted for a substantial portion of common variance (ECV = .520; ω_H_ = .724), suggesting moderate unidimensionality. Notably, the hyperactivity subscale retained reliable unique variance (ω_HS_ = .561), while inattention did not (ω_HS_ = .199), supporting selective interpretation of the former. These findings suggest that the ASRS-6 primarily captures general ADHD severity, with limited added value from inattention scores unless strong contextual reasons justify it (74, 75).

Practically, this supports the ASRS-6 total score as the primary index of ADHD symptoms in occupational or clinical screening, especially given its brevity and strong validity (8, 12). In Puerto Rico, Rosario-Hernández et al. (27) found that 3.3% of employed adults reported a prior ADHD diagnosis, reinforcing the tool’s relevance in workplace contexts. Studies show the ASRS-6 performs comparably to its full version (36), and its efficiency supports large-scale or time-sensitive screening efforts. Furthermore, research indicates that undiagnosed ADHD may increase workplace accidents (81), highlighting the importance of early detection paired with proper follow-up and confidentiality.

Beyond screening, evidence supports reasonable workplace accommodations (e.g., flexible scheduling, structured tasks) and skills-based interventions (e.g., metacognitive coaching) to support employees with ADHD (82, 83). Recent trials show that tailored workplace programs improve performance and executive functioning (84). A multimodal approach, combining screening, environmental adjustments, and individualized support, can improve employee well-being and productivity while reducing stigma.

### Limitations and future directions

This study, like many others, relied on self-report tools for ADHD identification, which may be biased due to under- or overreporting (e.g., 85). The relatively low proportion of participants with a self-reported ADHD diagnosis during the ROC analysis may have constrained screening accuracy estimates, as small sample sizes can generate imprecise and unstable ROC metrics, including sensitivity, specificity, and AUC (86–88). Furthermore, the study did not account for psychiatric comorbidities such as depression, anxiety, or substance use, conditions that frequently co-occur with ADHD and may confound ASRS-6 scores (89–92).

An additional limitation concerns the relatively low internal consistency of the Hyperactivity subscale (α = .65), which reflects the small number of items (two) and has been reported consistently in prior ASRS-6 validation studies. This level of reliability may limit the precision of inferences drawn from the hyperactivity scores when examined independently. However, consistent with the bifactor results observed in the present study, future research and applied use should prioritize interpretation of the ASRS-6 total score, which demonstrated stronger psychometric support and captured the majority of common variance across items (32, 73–75).

The cross-sectional design of the study also represents a limitation, as it precludes causal inferences regarding the relationships between ADHD symptoms and occupational or psychosocial outcomes (93, 94). Although the observed associations are theoretically consistent with prior longitudinal and clinical research, future studies using longitudinal or prospective designs are needed to examine temporal ordering, symptom stability, and potential reciprocal effects over time (95).

Cross-cultural validity also presents challenges. Although Lewczuk et al. (33) found that the ASRS-6 showed acceptable reliability across 42 countries, only partial invariance was achieved, and item functioning varied by culture. Cultural norms may influence symptom reporting, meaning that cut-offs might not generalize across regions. Validating the ASRS-6 in specific populations, such as Puerto Rican employees, is essential for accurate interpretation.

Issues of generalizability should be considered. Because the sample consisted exclusively of employed adults, the findings may not generalize to the broader population of working adults across different industries or employment conditions, nor to non-employed adults or clinical populations. Caution is therefore warranted when extending these results beyond the specific occupational and cultural context examined in this study (93, 96, 97).

Finally, little is known about the long-term stability of ADHD symptom structure in adulthood. While evidence supports childhood stability of inattention and hyperactivity (98), longitudinal adult data are scarce. The general factor may weaken or change over time, particularly in older adults. Long-term studies are needed to examine whether the ASRS-6 remains psychometrically stable across the adult lifespan, especially as ADHD-diagnosed cohorts age into later adulthood.

## Conclusions

In sum, the present study provides strong psychometric support for the use of the ASRS-6 as a brief, valid, and reliable tool for assessing ADHD symptoms in employed Spanish-speaking adults. The scale demonstrated a solid internal structure, invariance across key demographic groups, and excellent diagnostic accuracy, supporting its use in workplace wellness programs and occupational mental health screenings. Given the underdiagnosis of ADHD in adults and its documented impact on occupational functioning, validated brief screeners like the ASRS-6 offer an essential first step in identifying and supporting individuals at risk.

## Data Availability

The datasets used in this study are not publicly available because they contain sensitive information and are subject to ethical and confidentiality constraints approved by the Institutional Review Board. However, the data can be made available upon reasonable request to the corresponding author in the following email: erosario@psm.edu. Interested researchers may contact the corresponding author via email to discuss access to the dataset.

## References

[1] Scandar MG . Validity and reliability of the ASRS and WURS-25 scales for the diagnosis of attention deficit hyperactivity disorder in an Argentinian population. Rev Neurología. (2021) 72:77–84. doi: 10.33588/rn.7203.2019381, PMID: 33506485

[2] Faraone SV Banaschewski T Coghill D Zheng Y Biederman J Bellgrove MA . The World Federation of ADHD International Consensus Statement: 208 Evidence-based conclusions about the disorder. Neurosci Biobehav Rev. (2021) 128:789–818. doi: 10.1016/j.neubiorev.2021.01.022, PMID: 33549739 PMC8328933

[3] Kessler RC Adler L Barkley R Biederman J Conners CK Demler O . The prevalence and correlates of adult ADHD in the United States: results from the National Comorbidity Survey Replication. The American Journal of Psychiatry. (2006) 163:716–723. doi: 10.1176/ajp.2006.163.4.716, PMID: 16585449 PMC2859678

[4] Barkley RA Murphy KR . Impairment in occupational functioning and adult ADHD: The predictive utility of executive function (EF) ratings versus EF tests. Arch Clin Neuropsychol. (2010) 25:157–73. doi: 10.1093/arclin/acq014, PMID: 20197297 PMC2858600

[5] Adamou M Arif M Asherson P Aw TC Bolea B Coghill D . Occupational issues of adults with ADHD. BMC Psychiatry. (2013) 13:59. doi: 10.1186/1471-244X-13-59, PMID: 23414364 PMC3599848

[6] de Graaf R Kessler RC Fayyad J ten Have M Alonso J Angermeyer M . The prevalence and effects of adult attention-deficit/hyperactivity disorder (ADHD) on the performance of workers: results from the WHO World Mental Health Survey Initiative. Occup Environ Med. (2008) 65:835–42. doi: 10.1136/oem.2007.038448, PMID: 18505771 PMC2665789

[7] Halmøy A Fasmer OB Gillberg C Haavik J . Occupational outcome in adult ADHD: Impact of symptom profile, comorbid psychiatric problems, and treatment — a cross-sectional study of 414 clinically diagnosed adult ADHD patients. J Attention Disord. (2009) 13:175–87. doi: 10.1177/1087054708329777, PMID: 19372500

[8] Kessler RC Adler L Ames M Barkley RA Birnbaum H Greenberg P . The prevalence and effects of adult attention deficit/hyperactivity disorder on work performance in a nationally representative sample of workers. J Occup Environ Med. (2005) 47:565–72. doi: 10.1097/01.jom.0000166863.33541.39, PMID: 15951716

[9] American Psychiatric Association . Diagnostic and statistical manual of mental disorders (5th ed., text rev.; DSM-5-TR). (2022). doi: 10.1176/appi.books.9780890425787, PMID: 38300502

[10] Ramos-Quiroga JA Daigre C Valero S Bosch R Gómez-Barros N Nogueira M . Validation of the Spanish version of the attention deficit hyperactivity disorder adult screening scale (ASRS v. 1.1): A novel scoring strategy. Rev Neurología. (2009) 48:449–52. doi: 10.33588/rn.4809.2008677, PMID: 19396760

[11] Robles YI Saavedra JE Agüero YD . Propiedades psicométricas y métodos de medición de la escala de autoinforme sobre el trastorno de déficit de atención e hiperactividad en adultos: Tamizaje (ASRS-Tamizaje) en la población de Lima. Rev Neuro-Psiquiatría. (2020) 83:217–30. doi: 10.20453/rnp.v83i4.3887

[12] Ustun B Adler LA Rudin C Faraone SV Spencer TJ Berglund P . The world health organization adult attention-deficit/hyperactivity disorder self-report screening scale for DSM-5. JAMA Psychiatry. (2017) 74:520–7. doi: 10.1001/jamapsychiatry.2017.0298, PMID: 28384801 PMC5470397

[13] Daigre Blanco C Ramos-Quiroga JA Valero S Bosch R Roncero C Gonzalvo B . Adult ADHD Self-Report Scale (ASRS-v1.1) symptom checklist in patients with substance use disorders. Actas Españolas Psiquiatria. (2009) 37:299–305. Available online at: https://actaspsiquiatria.es/index.php/actas/article/view/1122., PMID: 20066581

[14] Lozano Ó.M Carmona J Muñoz-Silva A Fernández-Calderón F Díaz-Batanero C Sanchez-Garcia M . Adult ADHD self-report scale: comprehensive psychometric study in a spanish SUD sample. J Attention Disord. (2020) 24:1674–84. doi: 10.1177/1087054716664410, PMID: 27549779

[15] Pedrero Pérez EJ Puerta García C . ASRS v1.1. como instrumento de cribado del trastorno por deficit de atención e hiperactividad en adultos tratados por conductas adictivas: Propiedades psicométricas y prevalencia estimada. Adicciones. (2007) 19:393–408. doi: 10.20882/adicciones.298 18173102

[16] Sanchez-Garcia M Fernandez-Calderon F Carmona-Marquez J Chico-Garcia M Velez-Moreno A Perez-Gomez L . Psychometric properties and adaptation of the ASRS in a Spanish sample of patients with substance use disorders: Application of two IRT Rasch models. psychol Assess. (2015) 27:524–33. doi: 10.1037/pas0000064, PMID: 25580610

[17] Ariza Cruz CS . Evidencia de validez del Adult Self Report Scale (ASRS v1.1) en estudiantes universitarios de Lima Metropolitana. Ariza Cruz: Tesis de licenciatura, Universidad San Ignacio de Loyola (2019).

[18] Montiel-Nava C Ortiz León S Jaimes Medrano A González-Avila Z . Prevalencia del trastorno por déficit de atención-hiperactiviad en estudiantes universitarios venezolanos: Reporte preliminar. Investigación Clinica. (2012) 53:353–64. 23513486

[19] Boonstra AM Oosterlaan J Sergeant JA Buitelaar JK . Executive functioning in adult ADHD: A meta-analytic review. psychol Med. (2005) 35:1097–108. doi: 10.1017/S003329170500499X, PMID: 16116936

[20] Martel MM Nigg JT Schimmack U . Psychometrically InformedApproach to Integration of Multiple Informant Ratings in Adult ADHD in aCommunity-Recruited Sample. Assessment. (2017) 24:279–89. doi: 10.1177/1073191116646443, PMID: 27126924 PMC5085895

[21] Christian MS Bradley JC Wallace JC Burke MJ . (2009). Workplace safety: a meta-analysis of the roles of person and situation factors. The Journal ofapplied psychology. 94:1103–27. doi: 10.1037/a0016172, PMID: 19702360

[22] Halbesleben JR Wheeler AR Shanine KK . The moderating role of attention-deficit/hyperactivity disorder in the work engagement-performance process. Journal of occupational health psychology. (2013) 18:132–43. doi: 10.1037/a0031978, PMID: 23458061

[23] Kessler RC Lane M Stang PE Van Brunt DL . The prevalence and workplace costs of adult attention deficit hyperactivity disorder in a large manufacturing firm. Psychological medicine. (2009) 39:137–47. doi: 10.1017/S0033291708003309, PMID: 18423074

[24] Hodgkins P Montejano L Sasané R Huse D . Cost of illness and comorbidities in adults diagnosed with attention-deficit/hyperactivity disorder: A retrospective analysis. Primary Care Companion CNS Disord. (2011) 13:e1–e12. doi: 10.4088/PCC.10m01030, PMID: 21977356 PMC3184593

[25] Advokat C Martino L Hill BD Gouvier W . Continuous Performance Test (CPT) of college students with ADHD, psychiatric disorders, cognitive deficits, or no diagnosis. J Attention Disord. (2007) 10:253–60. doi: 10.1177/1087054706292106, PMID: 17242420

[26] Turjeman-Levi Y Itzchakov G Engel-Yeger B . Executive function deficits mediate the relationship between employees’ ADHD and job burnout. AIMS Public Health. (2024) 11:294–314. doi: 10.3934/publichealth.2024015, PMID: 38617412 PMC11007411

[27] Rosario-Hernández E Rovira Millán LV Santiago-Pacheco E Arzola-Berrios X Padovani CM Francesquini-Oquendo SM . ADHD and its effects on job performance: A moderated mediation model. Rev Caribeña Psicología. (2020) 4:1–25. doi: 10.37226/rcp.2020/01

[28] Fredriksen M Dahl AA Martinsen EW Klungsoyr O Faraone SV Peleikis DE . Childhood and persistent ADHD symptoms associated with educational failure and long-term occupational disability in adult ADHD. ADHD Attention Deficit Hyperactivity Disord. (2014) 6:87–99. doi: 10.1007/s12402-014-0126-1, PMID: 24497125 PMC4033786

[29] Barkley RA Murphy KR Fischer M . ADHD in adults: What the science says. New York, NY: The Guilford Press (2008).

[30] Demerouti E Bakker AB Nachreiner F Schaufeli WB . The job demands-resources model of burnout. J Appl Psychol. (2001) 86:499–512. doi: 10.1037/0021-9010.86.3.499 11419809

[31] Reyes Zamorano E Cárdenas Godínez EM García Vargas KL Aguilar Orozco NC Vázquez Medina J Díaz Flores A . Validación de constructo de la escala de autorreporte del Trastorno por Déficit de Atención con Hiperactividad (TDAH) en el adulto de la Organización Mundial de la Salud en población universitaria mexicana. Salud Ment. (2009) 32:343–50.

[32] Morin AJ Tran A Caci H . Factorial validity of the ADHD adult symptom rating scale in a french community sample: results from the chiP-ARD study. J attention Disord. (2016) 20:530–41. doi: 10.1177/1087054713488825, PMID: 23729493

[33] Lewczuk K Marcowski P Wizła M Gola M Nagy L Koós M . Cross-cultural adult ADHD assessment in 42 countries using the adult ADHD self-report scale screener. J Attention Disord. (2024) 28:512–30. doi: 10.1177/10870547231215518, PMID: 38180045

[34] Rosario-Hernández E Rovira Millán LV Velázquez Lugo Á Rodríguez N Cedeño A Cintrón E . Mediating role of job crafting and engagement in the relationship between ADHD and boredom at work on job performance, . Ponce, Puerto Rico: 4th Congress of Industrial-Organizational Psychology of Puerto Rico: Current Issues, Positive Vision, and Innovation, Ponce Hilton Hotel (2019).

[35] Adler LA Spencer T Faraone SV Kessler RC Howes MJ Biederman J . Validity of pilot Adult ADHD Self- Report Scale (ASRS) to Rate Adult ADHD symptoms. Ann Clin Psychiatry. (2006) 18:145–8. doi: 10.1080/10401230600801077, PMID: 16923651

[36] Brevik EJ Lundervold AJ Haavik J Posserud M-B . Validity and accuracy of the Adult Attention-Deficit/Hyperactivity Disorder (ADHD) Self-Report Scale (ASRS) and the Wender Utah Rating Scale (WURS) symptom checklists in discriminating between adults with and without ADHD. Brain Behav. (2020) 10:e01605. doi: 10.1002/brb3.1605, PMID: 32285644 PMC7303368

[37] Fayyad J Sampson NA Hwang I Adamowski T Aguilar-Gaxiola S Al-Hamzawi A . The descriptive epidemiology of DSM-IV Adult ADHD in the World Health Organization World Mental Health Surveys. Attention Deficit Hyperactivity Disord. (2017) 9:47–65. doi: 10.1007/s12402-016-0208-3, PMID: 27866355 PMC5325787

[38] Silverstein MJ Faraone SV Alperin S Biederman J Spencer TJ Adler LA . How informative are self-reported adult attention-deficit/hyperactivity disorder symptoms? An examination of the agreement between the adult attention-deficit/hyperactivity disorder self-report scale V1.1 and adult attention-deficit/hyperactivity disorder investigator symptom rating scale. J Child Adolesc Psychopharmacol. (2018) 28:339–49. doi: 10.1089/cap.2017.0082, PMID: 29172673

[39] Gray S Woltering S Mawjee K Tannock R . The Adult ADHD Self-Report Scale (ASRS): Utility in college students with attention-deficit/hyperactivity disorder. PeerJ. (2014) 2:e324. doi: 10.7717/peerj.324, PMID: 24711973 PMC3970798

[40] Schaufeli W Bakker A . Utrecht work engagement scale: Preliminary manual. Utrecht: Occupational Health Psychology Unit, Utrecht University (2003).

[41] Schaufeli WB Salanova M González-Romá V Bakker AB . The measurement of engagement and burnout: A confirmative analytic approach. J Happiness Stud. (2002) 3:7–92. doi: 10.1023/A:1015630930326

[42] Martínez Alvarado LY Rosario-Hernández E Rovira-Millán LV . La relación entre la inseguridad laboral y el bienestar psicológico en una muestra de asistentes de vuelo: El papel moderador del engagement en el trabajo. Cienc la Conducta. (2017) 32:99–127.

[43] Rodríguez-Montalbán R Martínez-Lugo M Sánchez-Cardona I . Análisis de las propiedades psicométricas de la Utrecht Work Engagement Scale en una muestra de trabajadores en Puerto Rico. Universitas Psychologica. (2014) 13:1255–1266. doi: 10.11144/Javeriana.UPSY13-4.appu

[44] Rosario-Hernández E Rovira-Millán LR . Desarrollo y validación de la Escala de Ciudadanía Organizacional. Revista Puertorriqueña de Psicología. (2004) 15:1–25.

[45] Rosario-Hernández E Rovira-Millán LR . Desarrollo y validación del Índice de Conductas Laborales Contraproducentes. Revista Interamericana de Psicología Ocupacional. (2008) 27:16–27.

[46] Martínez-Lugo M Rodríguez-Montalbán R . Cuando el trabajo aburre: Análisis de las propiedades psicométricas de la Escala de Aburrimiento Laboral (EAL). Revista Interamericana de Psicología Ocupacional. (2016) 35:7–20. doi: 10.21772/ripo.v35n1a01

[47] Rosario-Hernández E Rovira-Millán LV . Desarrollo y validación de una escala para medir actitudes hacia el retiro. Rev Puertorriqueña Psicología. (2002) 13:45–60.

[48] Rosario-Hernández E Rovira Millán LV Blanco-Rovira RA . Review of the psychometric properties of the Social Desirability Scale and the development of a short-form. Rev Caribeña Psicología. (2025) 9:e13313. doi: 10.37226/rcp.v9i1.13313

[49] Rosseel Y . lavaan: an R package for structural equation modeling. J Stat Software. (2012) 48:1–36. doi: 10.18637/jss.v048.i02

[50] Li CH . Confirmatory factor analysis with ordinal data: Comparing robust maximum likelihood and diagonally weighted least squares. Behav Res Methods. (2016) 48:936–49. doi: 10.3758/s13428-015-0619-7, PMID: 26174714

[51] Li C-H . The performance of ML, DWLS, and ULS estimation with robust corrections in structural equation models with ordinal variables. psychol Methods. (2016) 21:369–87. doi: 10.1037/met0000093, PMID: 27571021

[52] Howard MC Boudreaux M Cogswell J Manix KG Oglesby MT . A literature review of model fit and model comparisons with confirmatory factor analysis: Formalizing the informal in organizational science. Appl Psychol. (2025) 74:e12592. doi: 10.1111/apps.12592

[53] Kline RB . Principles and practice of structural equation modeling. New York, NY: The Guilford Press (2016).

[54] Marsh HW Balla JR Hau KT . An evaluation of incremental fit indexes: A clarification of mathematical and empirical properties. In: Marcoulides GA Schumacker RE , editors. Advanced structural equation modeling techniques. Lawrence Erlbaum, Mahwah, NJ (1996). p. 315–53.

[55] Byrne BM . Structural equation modeling with AMOS: Basic concepts, applications, and programming. New York, NY: Routledge (2016).

[56] Muthén LK Muthén BO . Mplus user’s guide. Los Angeles, CA: Muthén & Muthén (1998–2012).

[57] Wang J Wang X . Structural equation modeling applications using mplus. Chichester: John Wiley & Sons Ltd (2012). doi: 10.1002/9781118356258

[58] Cheung GW Rensvold RB . Evaluating goodness-of-fit indexes for testing measurement invariance. Struct Equation Modeling. (2002) 9:233–55. doi: 10.1207/S15328007SEM0902_5

[59] Rigdon EE . A necessary and sufficient identification rule for structural models estimated in practice. Multivariate Behav Res. (1995) 30:359–83. doi: 10.1207/s15327906mbr3003_4, PMID: 26789940

[60] Nunnally JC Bernstein IH . Psychometric theory. New York, NY: McGraw-Hill (1994).

[61] Hair JF Black WC Babin BJ Anderson RE . Multivariate data analysis. Hampshire, UK: Cengage Learning (2019).

[62] Fornell C Larcker DF . Evaluating structural equation models with unobservable variables and measurement error. J Marketing Res. (1981) 18:39–50. doi: 10.2307/3151312

[63] Chen FF . Sensitivity of goodness of fit indexes to lack of measurement invariance. Struct Equation Modeling. (2007) 14:464–504. doi: 10.1080/10705510701301834

[64] Hosmer DW Lemeshow S Sturdivant RX . Applied logistic regression. Hoboken, NJ: John Wiley & Sons (2013). doi: 10.1002/9781118548387

[65] Kessler RC Adler LA Gruber MJ Sarawate CA Spencer T Van Brunt DL . Validity of the World Health Organization Adult ADHD Self-Report Scale (ASRS) Screener in a representative sample of health plan members. Int J Methods Psychiatr Res. (2007) 16:52–65. doi: 10.1002/mpr.208, PMID: 17623385 PMC2044504

[66] Arias VB Ponce FP Núñez DE . Bifactor models of attention-deficit/hyperactivity disorder (ADHD): an evaluation of three necessary but underused psychometric indexes. Assessment. (2018) 25:885–97. doi: 10.1177/1073191116679260, PMID: 27872349

[67] Arildskov TW Virring A Lambek R Carlsen AH Sonuga-Barke EJS Østergaard SD . The factor structure of attention-deficit/hyperactivity disorder in schoolchildren. Res Dev Disabil. (2022) 125:104220. doi: 10.1016/j.ridd.2022.104220, PMID: 35462238

[68] Caci H Didier C Wynchank D . Validation and bifactor structure of the French Adult ADHD Symptoms Rating Scale v1.1 (ASRS). L’Encephale. (2024) 50:68–74. doi: 10.1016/j.encep.2022.11.007, PMID: 36641267

[69] Gomez R Kyriakides C Devlin E . Attention-deficit/hyperactivity disorder symptoms in an adult sample: Associations with Rothbart’s temperament dimensions. Pers Individ Dif. (2014) 60:73–8. doi: 10.1016/j.paid.2013.12.023

[70] Stanton K Forbes MK Zimmerman M . Distinct dimensions defining the Adult ADHD Self-Report Scale: Implications for assessing inattentive and hyperactive/impulsive symptoms. psychol Assess. (2018) 30:1549–59. doi: 10.1037/pas0000604, PMID: 29878817

[71] Vajsz K Paulina LR Miklósi M . The bifactor model of the Hungarian self-report version of the Strengths and Weaknesses of ADHD and Normal Behaviors scale. Eur Psychiatry. (2024) 67:S75–6. doi: 10.1192/j.eurpsy.2024.201 PMC11255780 39026526

[72] Bonifay W Lane SP Reise SP . Three concerns with applying a bifactor model as a structure of psychopathology. Clin psychol Sci. (2016) 5:184–6. doi: 10.1177/2167702616657069

[73] Reise SP . The rediscovery of bifactor measurement models. Multivariate Behav Res. (2012) 47:667–96. doi: 10.1080/00273171.2012.715555, PMID: 24049214 PMC3773879

[74] Reise SP Bonifay WE Haviland MG . Scoring and modeling psychological measures in the presence of multidimensionality. J Pers Assess. (2013) 95:129–40. doi: 10.1080/00223891.2012.725437, PMID: 23030794

[75] Rodriguez A Reise SP Haviland MG . Evaluating bifactor models: Calculating and interpreting statistical indices. psychol Methods. (2016) 21:137–50. doi: 10.1037/met0000045, PMID: 26523435

[76] Das D Cherbuin N Butterworth P Anstey KJ Easteal S . A population-based study of attention deficit/hyperactivity disorder symptoms and associated impairment in middle-aged adults. PloS One. (2012) 7:e31500. doi: 10.1371/journal.pone.0031500, PMID: 22347487 PMC3275565

[77] Fuermaier ABM Tucha L Butzbach M Weisbrod M Aschenbrenner S Tucha O . ADHD at the workplace: ADHD symptoms, diagnostic status, and work-related functioning. Journal of Neural Transmission. Vienna, Austria (2021) 128:1021–1031. doi: 10.1007/s00702-021-02309-z, PMID: 33528652 PMC8295111

[78] Eastwood JD Frischen A Fenske MJ Smilek D . The unengaged mind: Defining boredom in terms of attention. Perspect psychol Sci. (2012) 7:482–95. doi: 10.1177/1745691612456044, PMID: 26168505

[79] Malkovsky E Merrifield C Goldberg Y Danckert J . Exploring the relationship between boredom and sustained attention. Exp Brain Res. (2012) 221:59–67. doi: 10.1007/s00221-012-3147-z, PMID: 22729457

[80] Goh PK Lee CA Bansal PS Aguerrevere LE Rucker AT Martel MM . Interpretability and validity of a bifactor model of ADHD in young adults: Assessing the general “g” and specific IA and HI factors. J Psychopathol Behav Assess. (2020) 42:222–36. doi: 10.1007/s10862-019-09774-7

[81] Aydin M Eryilmaz A Bugrahan Gurcan M İyiler A Surucu S Yilmaz M . The impact of attention-deficit/hyperactivity disorder assessments on predicting occupational foreign body penetration injuries. Med Bull Haseki. (2025) 63:74–80. doi: 10.4274/haseki.galenos.2025.32042

[82] Hotte-Meunier A Sarraf L Bougeard A Bernier F Voyer C Deng J . Strengths and challenges to embrace attention-deficit/hyperactivity disorder in employment: A systematic review. Neurodiversity. (2024) 2:1–13. doi: 10.1177/27546330241287655

[83] Lauder K McDowall A Tenenbaum HR . A systematic review of interventions to support adults with ADHD at work-Implications from the paucity of context-specific research for theory and practice. Front Psychol. (2022) 13:893469. doi: 10.3389/fpsyg.2022.893469, PMID: 36072032 PMC9443814

[84] Grinblat N Rosenblum S . Work-MAP telehealth metacognitive work-performance intervention for adults with ADHD: randomized controlled trial. OTJR: Occupation Participation Health. (2023) 43:435–45. doi: 10.1177/15394492231159902, PMID: 36971429 PMC10336612

[85] Garcia-Rosales A Cortese S Vitoratou S . Measurement invariance of Attention Deficit/Hyperactivity Disorder symptom criteria as rated by parents and teachers in children and adolescents: A systematic review. PloS One. (2024) 19:e0293677. doi: 10.1371/journal.pone.0293677, PMID: 38394179 PMC10889893

[86] Berrar D Flach P . Caveats and pitfalls of ROC analysis in clinical microarray research (and hot to avoid them). Briefings Bioinf. (2012) 13:83–97. doi: 10.1093/bib/bbr008, PMID: 21422066

[87] Hajian-Tilaki K . Sample size estimation in diagnostic test studies of biomedical informatics. J Biomed Inf. (2014) 48:193–204. doi: 10.1016/j.jbi.2014.02.013, PMID: 24582925

[88] Hanczar B Hua J Sima C Weinstein J Bittner M Dougherty ER . Small-sample precision of ROC-related estimates. Bioinformatics. (2010) 26:822–30. doi: 10.1093/bioinformatics/btq037, PMID: 20130029

[89] Barbuti M Maiello M Spera V Pallucchini A Brancati GE Maremmani AGI . Challenges of treating ADHD with comorbid substance use disorder: considerations for the clinician. J Clin Med. (2023) 12:3096. doi: 10.3390/jcm12093096, PMID: 37176536 PMC10179386

[90] Choi WS Woo YS Wang SM Lim HK Bahk WM . The prevalence of psychiatric comorbidities in adult ADHD compared with non-ADHD populations: A systematic literature review. PloS One. (2022) 17:e0277175. doi: 10.1371/journal.pone.0277175, PMID: 36331985 PMC9635752

[91] Fu X Wu W Wu Y Liu X Liang W Wu R . Adult ADHD and comorbid anxiety and depressive disorders: a review of etiology and treatment. Front Psychiatry. (2025) 16:1597559. doi: 10.3389/fpsyt.2025.1597559, PMID: 40547117 PMC12179154

[92] Katzman MA Bilkey TS Chokka PR Fallu A Klassen LJ . Adult ADHD and comorbid disorders: clinical implications of a dimensional approach. BMC Psychiatry. (2017) 17:302. doi: 10.1186/s12888-017-1463-3, PMID: 28830387 PMC5567978

[93] Shadish WR Cook TD Campbell DT . Experimental and quasi-experimental designs for generalized causal inference. Houghton: Mifflin and Company (2002).

[94] Van der Stede WA . A manipulationist view of causality in cross-sectional survey research. J Accounting Literature. (2014) 33:567–80. doi: 10.1016/j.aos.2013.12.001

[95] Maxwell SE Cole DA . Bias in cross-sectional analyses of longitudinal mediation. psychol Methods. (2007) 12:23–44. doi: 10.1037/1082-989X.12.1.23, PMID: 17402810

[96] Rothwell PM . External validity of randomised controlled trials: “to whom do the results of this trial apply? Lancet. (2005) 365:82–93. doi: 10.1016/S0140-6736(04)17670-8, PMID: 15639683

[97] Tipton E . Improving generalizations from experiments using propensity score subclassification: Assumptions, properties, and contexts. J Educ Behav Stat. (2013) 38:239–66. doi: 10.3102/1076998612441947

[98] Leopold DR Christopher ME Olson RK Petrill SA Willcutt EG . Invariance of ADHD symptoms across sex and age: A latent analysis of ADHD and impairment ratings from early childhood into adolescence. J Abnormal Child Psychol. (2019) 47:21–34. doi: 10.1007/s10802-018-0434-6, PMID: 29691720 PMC6202270

